# Supplementary material for: Finding phylogeny-aware and biologically meaningful averages of metagenomic samples: L2UniFrac
Source: Bioinformatics. 2023 Jun 30;39(Suppl 1):i57–65. doi: 10.1093/bioinformatics/btad238 (PMC10311324; doi:10.1093/bioinformatics/btad238)
Supplement: btad238_Supplementary_Data [file btad238_supplementary_data.zip › btad238_Supplementary_Data/Koslicki.225.sup.clean.pdf]

Supplemental Materials: Finding phylogeny-aware and biologically meaningful averages of metagenomic samples: L_2UniFrac

Wei Wei[1], Andrew Millward[2], David Koslicki[1,2,3]

[1]Huck Institutes of Life Sciences, Pennsylvania State University
[2]Department of Computer Science and Engineering, Pennsylvania State University
[3]Department of Biology, Pennsylvania State University

March 23, 2023

S1 Mathematical descriptions and proofs

S1.1 Definitions and terminology

We recall the following definitions of L_1 and L_2 UniFrac from the Methods section of the main manuscript.

S1.1.1 L_1UniFrac

In the original description of UniFrac distance as 1-Wasserstein distance, each sample is represented as a probability vector in $\mathbb{R}^N$ where N is the number of nodes on the phylogenetic tree. Given such two vectors P and Q, the UniFrac distance between them can be computed by by computing

$$\text{UniFrac}(P, Q) = ||W(P - Q)||_{L_1}$$

where W is an $N \times N$ matrix with the i-th row being the indicator function describing the subtree rooted at node i scaled by length of the branch connecting node i and its ancestor (see section S1.2 below).

S1.1.2 L_2UniFrac

The L_2UniFrac can be expressed in a similar manner, with the L_1 norm replaced by L_2 norm and W scaled by the square-root of branch lengths instead. The scaling of branch lengths by taking square-root is motivated by the biological meaning that would result from this expression as noted by Evans and Matsen [2, 6]. Since the branch lengths are simply a measure of phylogenetic proximity, taking the square-root will not change the underlying principle of this computation. We denote this expression by

$$L_2\text{UniFrac}(P, Q) = ||W_{\sqrt{\cdot}}(P - Q)||_{L_2}. \tag{1}$$

For any distribution P, we will refer to the metric spaces where WP and $W_{\sqrt{\cdot}}P$ lie in the L_1UniFrac space and the L_2UniFrac space respectively.

S1.2 Invertibility of W and $W_{\sqrt{\cdot}}$

Claim 1. *For any given tree with non-zero branch lengths, the corresponding W or $W_{\sqrt{\cdot}}$ constructed as above is invertible.*

Proof. We proof this claim by showing that W and $W_{\sqrt{\cdot}}$ are lower triangular matrices with non-zero diagonal entries, indicating that they are full-rank matrices and are thus invertible.

To construct W or $W_{\sqrt{\cdot}}$ given a tree T with N nodes, we first define an indicator function as follows:

$$w_j(i) = \begin{cases} 1 & \text{if } i \text{ is a node on the subtree of } T \text{ rooted at node } j \\ 0 & \text{otherwise.} \end{cases} \tag{2}$$

Let w_j be a row vector with entry i being $w_j(i)$ for $1 \leq i \leq n$. Then w_j will have 1 on position j as well as all descendants of j, and 0 everywhere else. In particular, $w_j(i) = 0 \; \forall i \geq j$. Let W' be a matrix with row j being w_j for $1 \leq j \leq n$. Then it can be observed that W' is an $N \times N$ lower-triangular matrix with 1 on the diagonal, and is thus invertible. Now W (or $W_{\sqrt{\cdot}}$) is constructed by scaling W' row by row by a positive factor (some non-zero function of the length of the corresponding branch, be it the branch length itself or the square-root of it). The resulting matrix is still a lower-triangular matrix with non-zero diagonal entries, and is hence invertible. □

In the next section, we present the algorithms that compute $W_{\sqrt{\cdot}}$ and its inverse respectively, and the utilities of these operations.

S1.3 Motivation and computations of $W_{\sqrt{\cdot}}$ and $W_{\sqrt{\cdot}}^{-1}$

The first motivation for the computation of $W_{\sqrt{\cdot}}$ is for the purpose of clustering in L_2UniFrac space, as demonstrated in Section 3.1. As the definition (1) of L_2UniFrac shows, the L_2UniFrac distance between two distribution vectors P and Q can be equivalently expressed as $||W_{\sqrt{\cdot}}P - W_{\sqrt{\cdot}}Q||_{L_2}$, which is simply the L_2 distance between two $W_{\sqrt{\cdot}}$-transformed vectors. Instead of constructing the actual matrix $W_{\sqrt{\cdot}}$ and perform matrix multiplication, which is both time-consuming and space-consuming, Algorithm 1 performs this exact process in a bottom-up fashion with respect to tree T, resulting in a linear-time and linear-space performance. As this algorithm can be perceived as aggregating the masses from leaves up the tree T, resulting in an 'aggregated vector' in L_2UniFrac space, we term this algorithm 'L_2-aggregate' to illustrate this property. The correctness of a very similar algorithm, but the L_1UniFrac case, is shown by Wei and Koslicki in [13], which can be directly adopted to demonstrate the correctness of Algorithm 1 by substituting $l(i)$ with $\sqrt{l(i)}$.

Algorithm 1 L_2-aggregate: an algorithm to obtain the L_2-aggregated vector given a probability vector P

Input:
P, T, where P is a probability vector with entries representing relative abundances summing up to 1, T being the phylogenetic tree with the ancestor of a node i denoted by $a(i)$ and the branch length between i and $a(i)$ denoted by $l(i)$.
Initialization: $\overline{P} = P$
for i in $1, \ldots, |T| - 1$ **do** ▷ Ordered from the leaves to the root
    $\overline{P}[a(i)] += \overline{P}[i]$ ▷ aggregating mass up
    $\overline{P}[i] = \overline{P}[i] \cdot \sqrt{l(i)}$
end for
return $\overline{P}$

Similarly, we have Algorithm 2 that computes $W_{\sqrt{\cdot}}^{-1}$, which we termed 'inverse L_2-aggregate' as it reverses the effect of Algorithm 1 to obtain the original vector P from its aggregated counterpart $\overline{P}$.

Algorithm 2 Inverse-aggregate: an algorithm that reverse L_2-aggregate to obtain a probability vector in the original space, given an aggregated vector in the L_2UniFrac space.

Input:
$\overline{P}$, T, where $\overline{P}$ is a probability vector in the L_2UniFrac space, T being the taxonomic tree with the ancestor of a node i denoted by $a(i)$ and the branch length between i and $a(i)$ denoted by $l(i)$.
Initialization: $P = \overline{P}$
for i in $1, \ldots, |T| - 1$ **do** ▷ Ordered from the leaves to the root
    $v = \overline{P}[i]$
    $P[a(i)] -= \frac{1}{\sqrt{l(i)}} * v$
end for
return P

It is noteworthy that the existence of Algorithm 2 is not merely for mathematical completeness, but has the actual application of converting an aggregated vector $\overline{P}$ in the L_2UniFrac space to the corresponding distribution vector P in the distribution space, even if P is not a naturally-existing distribution by itself.

For instance, $\overline{P}$ can be a vector that is obtained by applying Algorithm 1 to a group of samples belonging to the same environment and taking the average of these L_2-aggregated vectors. Algorithm 2 allows one to obtain the corresponding distribution P, which can be seen as an 'average' distribution representing the environment.

S1.4 Obtaining the average sample in L_1 and L_2 UniFrac space

We first give a formal definition to average distribution in a metric space.

Definition 1 (Barycenter)**.** Let $X = \left(\mathbb{R}^N, d\right)$ be a metric space, the barycenter x^*, or the average distribution of n probability distributions x^1, x^2 ..., x^n in X is defined by:

$$x^* = \arg\min_x \sum_{i=1}^{n} d(x^i, x). \tag{3}$$

Claim 2. *The barycenter of a finite number of vectors in L_1 space is equivalent to the median of the vectors.*

Proof. Let x^1, x^2, ..., $x^n \in (\mathbb{R}^N, |.|)$ be n vectors in L_1 space, where $|.|$ is the L_1-norm. Then the barycenter x^* satisfies

$$x^* = \arg\min_x \sum_{i=1}^{n} |x - x^i| \tag{4}$$

$$\Rightarrow x_j^* = \arg\min_{x_j} \sum_{i=1}^{n} |x_j - x_j^i| \quad \forall 1 \leq j \leq N. \tag{5}$$

$$\tag{6}$$

Suppose x_j^* is not the median of $x_j^1, x_j^2, ..., x_j^n$. Then, assume without loss of generality that p out of n x_j's are smaller than x_j^* and q out of n x_j's are greater than or equal to x_j^*, such that $p + q = n$ and $p < q$. Let $\overline{x}_j$ be the smallest number greater than x^* and let $d = |\overline{x}_j - x_j^*|$. Then

$$\sum_{i=1}^{n} |\overline{x}_j - x_j^i| = \sum_{i=1}^{n} |x_j^* - x_j^i| + pd - qd < \sum_{i=1}^{n} |x_j^* - x_j^i| \tag{7}$$

which is a contradiction. □

S1.4.1 The insufficiency of L_1 average distribution

The result of the previous section shows that, given n distributions P^1, P^2, ..., P^n and aggregation matrix W, the barycenter of WP^1, WP^2, ..., WP^n in L_1UniFrac space is equivalent to their component-wise median. However, when this median vector is projected back to the distribution space by applying W^{-1}, it does not guarantee a biologically meaningful average distribution with respect to L_1UniFrac. We provide an example of this below.

Example 1. *Consider* $P^1 = (0.3, 0, 0, 0.7)$*,* $P^2 = (0, 0.3, 0.2, 0.5)$*,* $P^3 = (0.4, 0.4, 0.2, 0)$ *and* $W = \begin{bmatrix} .9 & 0 & 0 & 0 \\ 0 & .9 & 0 & 0 \\ .1 & .1 & .1 & 0 \\ 1 & 1 & 1 & 1 \end{bmatrix}$.

We have $WP^1 = (0.27, 0, 0.03, 1)$, $WP^2 = (0, 0.27, 0.05, 1)$, $WP^3 = (0.36, 0.36, 0.1, 1)$ and the barycenter $WP^* = (0.27, 0.27, 0.05, 1)$. However, $W^{-1}P^* = (0.3, 0.3, -0.1, 0.5)$, which contains a negative entry, despite the fact that the entries sum up to 1. This means that the $W^{-1}P^*$ fails to uphold the biological meaning of representing an average microbial distribution.

This issue does not only occur in carefully constructed theoretical examples, but is actually prevalent in real world cases. See Table 1 of the main text where this same problem occurs in every real-world environment we studied.

This issue can be avoided when L_2UniFrac is used instead of L_1UniFrac.

S1.4.2 The advantages of L_2 average distribution

Claim 3. *The barycenter of a finite number of vectors in L_2 space is equivalent to component-wise mean of the vectors.*

Proof. Let x^1, x^2, ..., $x^n \in (\mathbb{R}^N, ||.||)$ be n vectors in L_2 space, where $||.||$ is the L_2-norm. Then the barycenter x^* satisfies

$$x^* = \arg\min_x \sum_{i=1}^{n} \sqrt{\sum_{j=1}^{N} (x_j^i - x_j)^2} \tag{8}$$

$$\Rightarrow x_j^* = \arg\min_x \sum_{i=1}^{n} (x_j^i - x_j)^2 \tag{9}$$

This implies $\sum_{i=1}^{n}(x_j^i - x_j)^2$ attains its minimum when $x_j = x_j^*$. Taking the derivative of $\sum_{i=1}^{n}(x_j^i - x_j^*)^2$, we have

$$\sum_{i=1}^{n} (x_j^i - x_j^*) = 0 \tag{10}$$

$$\sum_{i=1}^{n} x_j^i - n x_j^* = 0 \tag{11}$$

$$x_j^* = \frac{1}{n} \sum_{i=1}^{n} x_j^i. \tag{12}$$

$$\tag{13}$$

□

Claim 4. *Given n probability vectors $x^1, x^2, ..., x^n$ in $\mathbb{R}^N$ satisfying $x_j^i \geq 0\ \forall 1 \leq j \leq N$ and $\sum_{j=1}^{N} x_j^i = 1\ \forall 1 \leq i \leq n$, and an invertible matrix $W \in \mathbb{R}^{\mathbb{N}\times\mathbb{N}}$, let x^* denote the L_2-average vector obtained as described above by successively applying W, taking the average, and applying W^{-1}. Then x^* is a probability vector. Namely, $x_j^* \geq 0\ \forall 1 \leq j \leq N$ and $\sum_{j=1}^{N} x_j^* = 1$.*

Proof. Let $\overline{x}$ denote the mean vector of the L_2-aggregated vectors in the L_2UniFrac space after applying W. Namely,

$$\overline{x} = \frac{1}{n} \sum_{i=1}^{n} W x^i \tag{14}$$

$$\Rightarrow \overline{x}_j = \frac{1}{n} \sum_{i=1}^{n} (w_{j1} x_1^i + w_{j2} x_2^i + ... + w_{jN} x_N^i) \tag{15}$$

$$= \frac{1}{n} (w_{j1} \sum_{i=1}^{n} x_1^i + w_{j2} \sum_{i=1}^{n} x_2^i + ... + w_{jN} \sum_{i=1}^{n} x_N^i).$$

Since W is invertible, there exists W^{-1} with k,l-entry denoted by w'_{kl} such that $\overline{W} := W^{-1}W$ satisfies

$$\overline{w}_{kl} = \sum_{j=1}^{N} w'_{kj} w_{jl} = \begin{cases} 1 & \text{if } k = l \\ 0 & \text{otherwise} \end{cases}. \tag{16}$$

By definition,

$$x^* = W^{-1}\overline{x} \tag{17}$$

$$\Rightarrow x_j^* = (w'_{j1}\ w'_{j2}\ ...\ w'_{jN}) \cdot \overline{x}$$

$$= \frac{1}{n}(w'_{j1}\ w'_{j2}\ ...\ w'_{jN}) \cdot \begin{bmatrix} \sum_{j=1}^{N}(w_{1j}\sum_{i=1}^{n} x_1^i) \\ \sum_{j=1}^{N}(w_{2j}\sum_{i=1}^{n} x_2^i) \\ \vdots \\ \sum_{j=1}^{N}(w_{Nj}\sum_{i=1}^{n} x_N^i) \end{bmatrix}$$

$$= \frac{1}{n}(\sum_{k=1}^{N} w'_{jk} \sum_{j=1}^{N} w_{1j} \sum_{i=1}^{n} x_k^i)$$

$$= \frac{1}{n}(\sum_{i=1}^{n} x_1^i \sum_{k=1}^{N} w'_{jk}w_{k1} + \sum_{i=1}^{n} x_2^i \sum_{k=1}^{N} w'_{jk}w_{k2} + ... + \sum_{i=1}^{n} x_N^i \sum_{k=1}^{N} w'_{jk}w_{kN})$$

$$= \frac{1}{n}\sum_{i=1}^{n} x_j^i \sum_{k=1}^{N} w'_{jk}w_{kj} \text{ by (16)}$$

$$= \frac{1}{n}\sum_{i=1}^{n} x_j^i. \tag{18}$$

Since $x_j^i \geq 0 \forall i, j$, $x_j^* \geq 0 \forall j$. Also, $\sum_{j=1}^{N} x_j^* = \frac{1}{n}\sum_{i=1}^{n}\sum_{j=1}^{N} x_j^i = \frac{1}{n}(n) = 1$. □

This guarantees that the barycenter obtained with respect to L_2UniFrac will always be a distribution. Next, we show that not only is this x^* an distribution, it is also the barycenter of x^1 through x^n in $(\mathbb{R}^N, d)$ where d is the L_2UniFrac metric.

Claim 5. *Given probability distributions x^1, x^2, ..., x^n in $\mathbb{R}^N$ and aggregation matrix W, let y be the barycenter of Wx^1, Wx^2, ..., Wx^n in L_2 UniFrac space. Then $W^{-1}y$ is the barycenter of x^1, x^2, ..., x^n in the distribution space under the L_2 UniFrac metric.*

Proof. Let x^* be the barycenter of x^1, x^2, ..., x^2 under L_2UniFrac.Then

$$x^* = \arg\min_x \sum_{i=1}^{n} ||W(x^i - x)|| \tag{19}$$

$$= \arg\min_x \sum_{i=1}^{n} ||W(x^i - x)||^2 \tag{20}$$

$$= \arg\min_x \sum_{i=1}^{n}\sum_{j=1}^{N}(\sum_{k=1}^{N} w_{jk}(x_k^i - x_k))^2 \tag{21}$$

$$= \arg\min_x \sum_{i=1}^{n}(\sum_{k=1}^{N} w_{jk}(x_k^i - x_k))^2 \quad \forall 1 \leq j \leq N \tag{22}$$

$$\Rightarrow \sum_{i=1}^{n}\sum_{k=1}^{N} w_{jk}(x_k^i - x_k^*) = 0 \tag{23}$$

$$\sum_{k=1}^{N} w_{jk}(\sum_{i=1}^{n} x_k^i - nx_k^*) = 0. \tag{24}$$

where the derivative is taken in line (23) and is known to be zero as it's at a minimum. Since W is an arbitrary aggregation matrix, it has to be the case that $\sum_{i=1}^{n} x_k^i = nx_k^*$. Which in turn implies that $x_k^* = \frac{1}{n}\sum_{i=1}^{n} x_k^i$ for all k. By claim 4, $x^* = W^{-1}y$. □

Moreover, (18) shows that the L_2-average distribution obtained in this way is equivalent to taking the L_2 mean of the original distribution component-wise. This nice property is again absent in L_1UniFrac, as can be seen in this simple counter-example of $P^1 = (1, 0, 0)$, $P^2 = (0.17, 0.33, 0.5)$ and $P^3 = (0.33, 0.5, 0.17)$, of which the component-wise median equates to $(0.33, 0.33, 0.17)$, which does not have components summing up to 1.

S1.5 Barycenter with respect to L_2UniFrac metric

In this paper, we introduced the use of L_2UniFrac metric in place of the 1-Wasserstein-equivalent L_1UniFrac, and discussed its biological advantage of being able to yield the average distribution. In this section, we will discuss the significance of our results in a broader perspective.

Despite its popularity in measuring the dissimilarity between probability distributions, the Wasserstein distance is difficult to compute in general, having a cubic time complexity with respect to the number of supports [12]. Various attempts have been made to reduce the computational cost, either through approximation [10, 7], or projection to lower dimensional space [8], or looking for special cases where the computation can be simplified, such as the case of tree-Wasserstein distance [3]. We give a brief description of tree-Wasserstein distance.

Definition 2 (Tree metric)**.** Given a finite set Ω, a metric $d : \Omega \times \Omega \to \mathbb{R}$ is a tree metric if there exists a tree T with non-negative edge lengths such that any element in Ω can be represented as a node on T and for any $x, y \in \Omega$, $d(x, y)$ can be represented as a (unique) path between nodes x and y on T. [3]

A tree-Wasserstein metric is simply a measurement of the optimal transport with a the underlying metric being a tree metric. Under these definitions, it can be noticed that the (L_1) UniFrac metric, being equivalent to the 1-Wasserstein distance, is specifically a tree-Wasserstein metric with the phylogenetic tree being the underlying tree structure. The tree-Wasserstein metric is a special case of the Wasserstein metric due to the fact that it has a closed a form and can thus be computed in linear time [5, 3, 12].

Another important question related to the Wasserstein metric is finding the barycenter in a Wasserstein metric space. The Wasserstein barycenter has been a widely studied topic and finds its application in diverse fields such as natural language processing [14], model ensembling [1], and image processing [11, 9]. As can be expected, the computation of Wasserstein barycenter is closely related to the computation of the Wasserstein metric itself. Given the relative ease of computing tree-Wasserstein metrics, there has been a fair amount of effort invested in studying tree-Wasserstein barycenters. Though various methods have been proposed to speed up the computation of tree-Wasserstein barycenters, finding the exact barycenters remain a hard problem [12]. Most of the proposed methods still involve complicated algorithms and constrains, and are approximations in nature [12, 4]. Similarly for the case of L_1UniFrac, efficient computation of the exact barycenter remains an open problem, as demonstrated in Section S1.4.1. However, by simply changing the metric from L_1UniFrac to L_2UniFrac, we are able to obtain the exact barycenter with respect to this metric by simply taking the component-wise mean.

S2 Supplementary figures

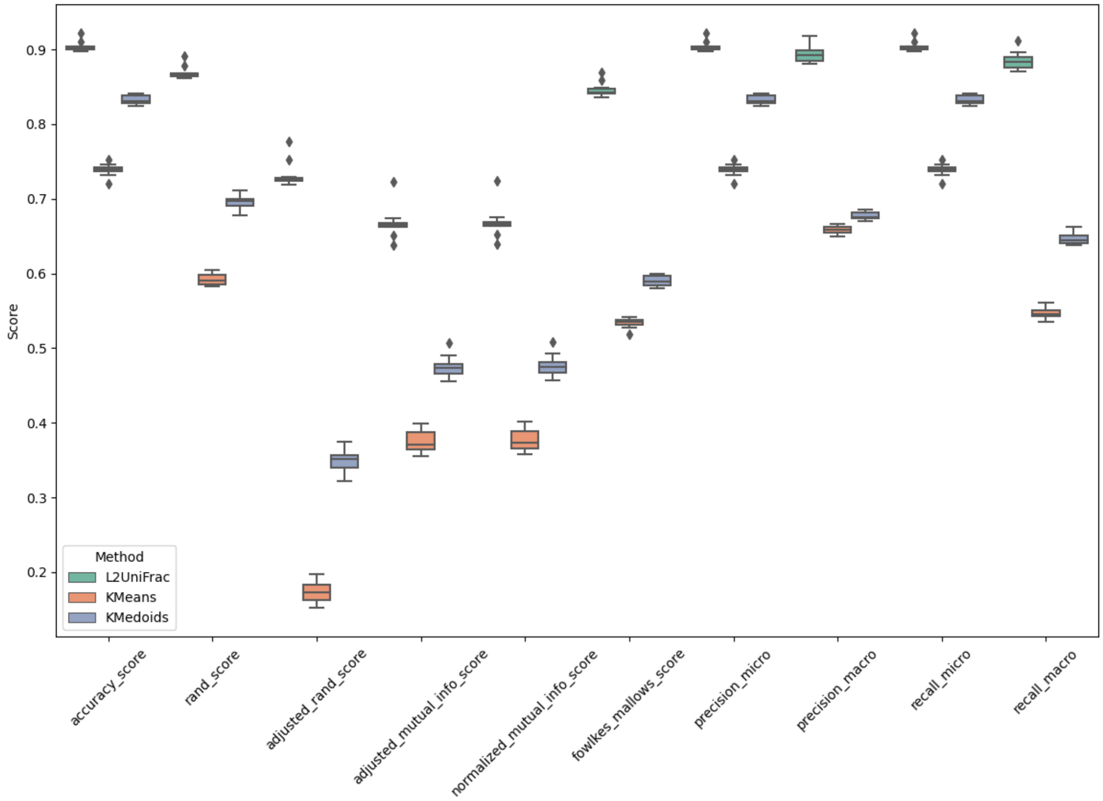

Figure S1: Other scores evaluating classification performance. Higher score indicates a better performance in all cases.

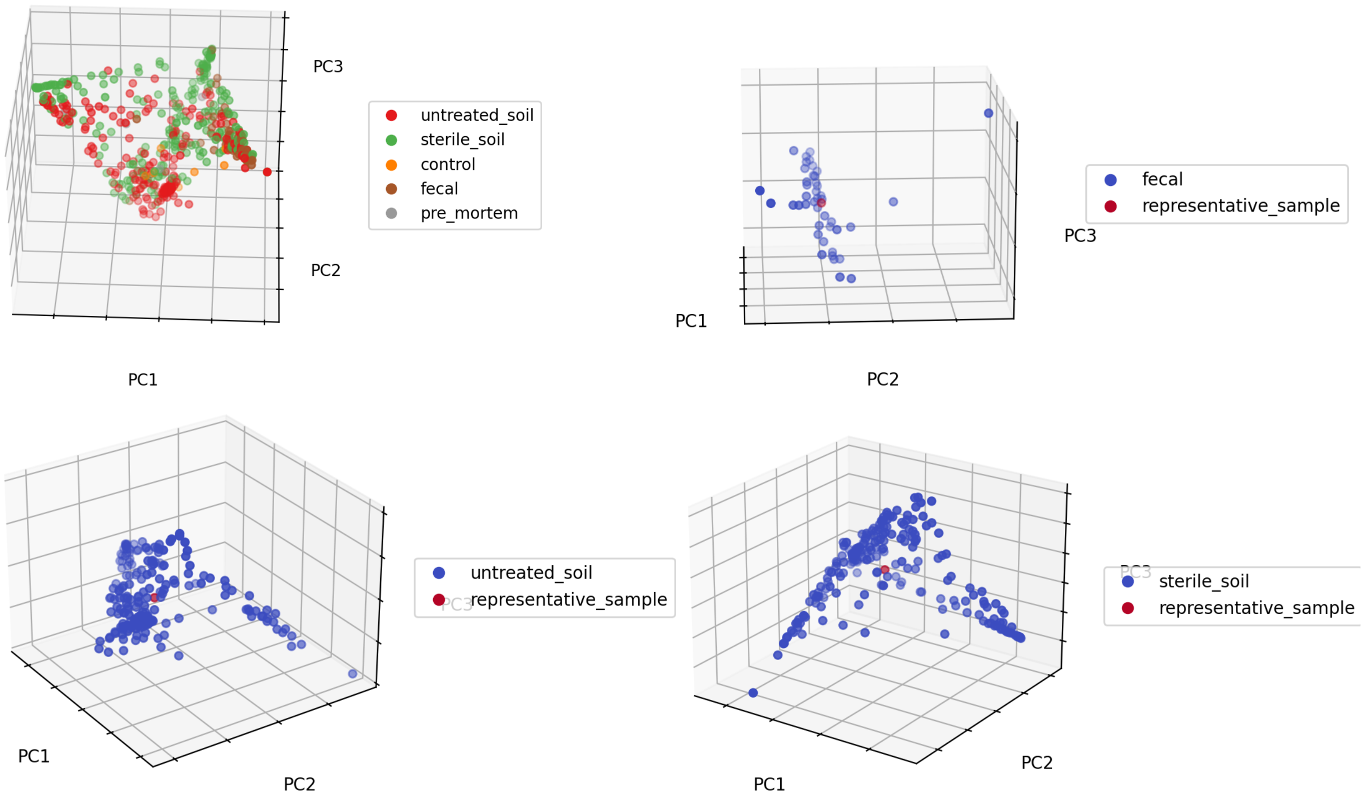

Figure S2: PCoA plots showing the representative samples with respect to all the samples from the respective environments.

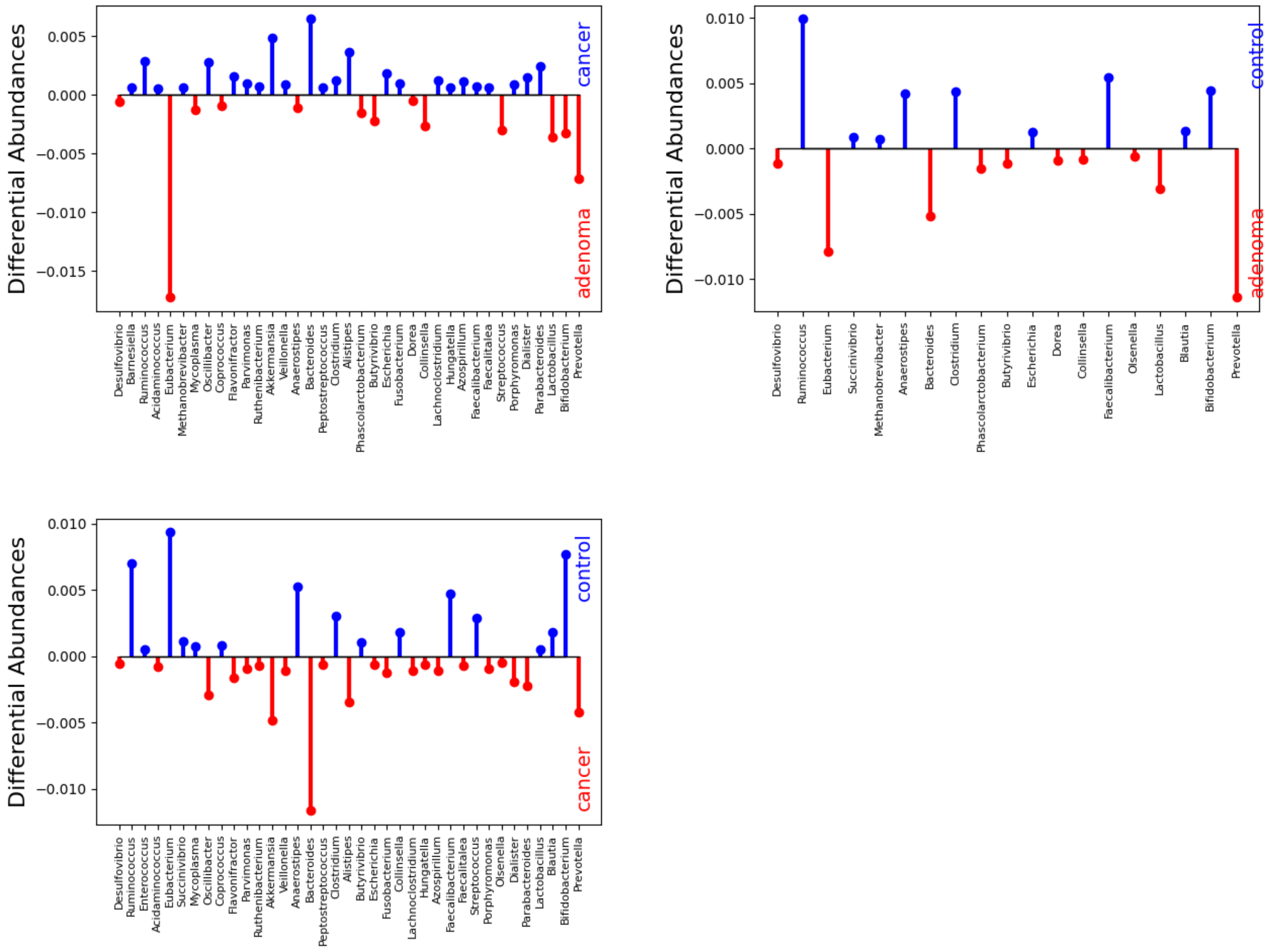

Figure S3: Differentially abundant genuses between each of the condition pairs. The values are measured in terms of the L_2UniFrac.

References

[1] Pierre Dognin et al. "Wasserstein Barycenter Model Ensembling". In: *arXiv* (2019). DOI: 10.48550/arxiv.1902.04999.

[2] Steven N. Evans and Frederick A. Matsen. "The phylogenetic Kantorovich–Rubinstein metric for environmental sequence samples". In: *Journal of the Royal Statistical Society: Series B (Statistical Methodology)* 74.3 (2012), pp. 569–592. ISSN: 1467-9868. DOI: 10.1111/j.1467-9868.2011.01018.x.

[3] Tam Le et al. "Tree-Sliced Variants of Wasserstein Distances". In: *arXiv* (2019). DOI: 10.48550/arxiv.1902.00342.

[4] Tam Le et al. "Tree-Wasserstein Barycenter for Large-Scale Multilevel Clustering and Scalable Bayes". In: *arXiv* (2019). DOI: 10.48550/arxiv.1910.04483.

[5] Catherine Lozupone and Rob Knight. "UniFrac: a New Phylogenetic Method for Comparing Microbial Communities". In: *Applied and Environmental Microbiology* 71.12 (2005), pp. 8228–8235. ISSN: 0099-2240. DOI: 10.1128/aem.71.12.8228-8235.2005.

[6] Jason McClelland. "Wasserstein B-diversity metrics over graphs: Derivation, efficient computation and applications". PhD thesis. 2018.

[7] Gabriel Peyre and Marco Cuturi. "Computational Optimal Transport". In: *Foundations and Trends in Machine Learning* 11.5-6 (2019), pp. 355–607.

[8] Julien Rabin et al. "Scale Space and Variational Methods in Computer Vision, Third International Conference, SSVM 2011, Ein-Gedi, Israel, May 29 – June 2, 2011, Revised Selected Papers". In: *Lecture Notes in Computer Science* (2012), pp. 435–446. ISSN: 0302-9743. DOI: 10.1007/978-3-642-24785-9_37.

[9] Julien Rabin et al. "Scale Space and Variational Methods in Computer Vision, Third International Conference, SSVM 2011, Ein-Gedi, Israel, May 29 – June 2, 2011, Revised Selected Papers". In: *Lecture Notes in Computer Science* (2012), pp. 435–446. ISSN: 0302-9743. DOI: 10.1007/978-3-642-24785-9_37.

[10] Ludger Rüschendorf. "The Wasserstein distance and approximation theorems". In: *Probability Theory and Related Fields* 70.1 (1985), pp. 117–129. ISSN: 0178-8051. DOI: 10.1007/bf00532240.

[11] Dror Simon and Aviad Aberdam. "Barycenters of Natural Images - Constrained Wasserstein Barycenters for Image Morphing". In: *2020 IEEE/CVF Conference on Computer Vision and Pattern Recognition (CVPR)* 00 (2020), pp. 7907–7916. DOI: 10.1109/cvpr42600.2020.00793.

[12] Yuki Takezawa et al. "Fixed Support Tree-Sliced Wasserstein Barycenter". In: *arXiv* (2021). DOI: 10.48550/arxiv.2109.03431.

[13] Wei Wei and David Koslicki. "WGSUniFrac: Applying UniFrac Metric to Whole Genome Shotgun Data". In: *22nd International Workshop on Algorithms in Bioinformatics (WABI 2022)*. Ed. by Christina Boucher and Sven Rahmann. Vol. 242. Leibniz International Proceedings in Informatics (LIPIcs). Dagstuhl, Germany: Schloss Dagstuhl – Leibniz-Zentrum für Informatik, 2022, 15:1–15:22. ISBN: 978-3-95977-243-3. DOI: 10.4230/LIPIcs.WABI.2022.15. URL: https://drops.dagstuhl.de/opus/volltexte/2022/17049.

[14] Hongteng Xu et al. "Distilled Wasserstein Learning for Word Embedding and Topic Modeling". In: *arXiv* (2018). DOI: 10.48550/arxiv.1809.04705.
